# Urinary iodine in early pregnancy is associated with subclinical hypothyroidism in Tianjin, China: an observational study

**DOI:** 10.1186/s12902-017-0162-x

**Published:** 2017-02-17

**Authors:** Kunling Wang, Jie Zhang, Fengao Li, Wanqi Zhang, Hao Wang, Li Ding, Yaxin Liu, Laixiang Lin, Shuang Zhang, Mei Zhu

**Affiliations:** 1 Department of Endocrinology and Metabolism, Tianjin Medical University General Hospital, No154 Anshan Road, Heping District, Tianjin, 300052 China; 2 Department of Surgery, Tianjin Medical University General Hospital, No154 Anshan Road, Heping District, Tianjin, 300052 China; 3 Department of Nutrition and Food Hygiene, School of Public Health, Tianjin Medical University, Tianjin, 300070 China; 4 Key Lab of Hormone and Development, Institute of Endocrinology, Tianjin Medical University, Tianjin, 300070 China; 5 Project Office, Tianjin Women’s and Children’s Health Center, No96 Guizhou Road, Heping District, Tianjin, 300070 China

**Keywords:** Early pregnancy, Subclinical hypothyroidism, Urine iodine concentration, Anti-thyroid peroxidase antibody (TPOAb), Anti-thyroid globulin antibody (TPOAb), Idiopathic subclinical hypothyroidism

## Abstract

**Background:**

Subclinical hypothyroidism (SH) is associated with adverse obstetric outcomes and neurodevelopment disorders. Both iodine deficiency and excess are associated with SH; however, few data regarding iodine nutrition status of pregnant women with SH are available. This study aimed to clarify whether iodine deficiency or excess is associated with SH, especially, when test results for anti-thyroid autoantibodies are negative.

**Methods:**

A total of 115 women with SH and 104 women with euthyroidism (EH) in early pregnancy in Tianjin, China were investigated, and their serum thyroid-stimulating hormone, free thyroxine, free triiodothyronine, anti-thyroid peroxidase antibody (TPOAb), anti-thyroid globulin antibody (TGAb), urinary iodine (UIC), and urinary creatinine (UCr) concentrations were measured. Thyroid ultrasonography was performed to determine thyroid echogenicity and volume. The UIC, UIC/UCr ratio, prevalence of TPOAb and TGAb positivity, and thyroid gland volume were compared between the EH and SH groups. UIC and ultrasonographic features were analysed in subjects in the SH group who were negative for TPOAb and TGAb.

**Results:**

Median UIC of SH (154.0 μg/L) and EH (150.1 μg/L) met the World Health Organization criterion for iodine sufficiency in pregnant women. Neither UIC nor the UIC/UCr ratio differed significantly between groups. The prevalence of TPOAb and TGAb positivity in the SH group was significantly higher than that in the EH group (*P* < 0.01). The percentage of subjects with UIC ≥ 250 μg/L in the SH group was significantly higher than that in the EH group (*p* = 0.004). The percentage of subjects negative for autoantibodies and UIC ≥ 250 μg/L in the SH group tended to be higher than that in subjects in the EH group negative for autoantibodies, but the difference was not statistically significant (*p* = 0.025, adjusted test level α = 0.0167). Eight of 18 subjects in the SH group with negative results for TPOAb and TGAb were diagnosed with Hashimoto thyroiditis by means of thyroid ultrasonography.

**Conclusions:**

Women in early pregnancy with SH in Tianjin were iodine sufficient, but still at risk of iodine deficiency as pregnancy progressed. UIC ≥ 250 μg/L was associated with increased risk of SH. Serological negative autoimmune thyroiditis and UIC ≥ 250 μg/L may play a role in pathogenesis of SH cases with negative results for autoantibodies.

## Background

Hypothyroidism is a common comorbidity of pregnancy and can be further categorised as clinical or subclinical hypothyroidism (SH) according to reduced or normal free thyroxine (FT_4_) levels. Clinical hypothyroidism can result in retarded foetal growth, mental development disorders, and adverse obstetric outcomes. Mounting clinical evidence suggests that SH contributes to an increased rate of obstetric complications and adverse birth outcomes including gestational diabetes, gestational hypertension, miscarriage, premature birth, small-for-gestational-age babies, placental abruption, and postpartum haemorrhage [1–4]. It is estimated that the prevalence of SH in pregnancy is 2.0–2.5% [5] worldwide and 4% [6] in China.

Iodine is the most important trace element for thyroid hormone synthesis, and both iodine deficiency and iodine excess are associated with SH [7, 8]. There was widespread iodine deficiency in China until universal salt iodisation in 1995. Since then, the prevalence of iodine deficiency diseases has decreased remarkably, while the prevalence of autoimmune thyroiditis and SH increased [9]. Pregnant women are vulnerable to iodine deficiency, and some cross-sectional surveys in different provinces and areas of China have showed iodine deficiency in pregnancy [10, 11]. However, few data regarding iodine nutrition status in pregnant women with SH are available. An epidemic investigation in Tianjin, a northern coastal city in China that is abundant in marine products, revealed iodine sufficiency [12]. To determine the iodine nutrition status and the prevalence of positivity for anti-thyroid peroxidase antibody (TPOAb) and anti-thyroid globulin antibody (TGAb) in early pregnancy with SH in Tianjin, this study evaluated the urinary iodine concentration (UIC), TPOAb and TGAb positivity, and ultrasonographic features of the thyroid gland in early pregnancy in relation to SH, considering evidence regarding iodine supplementation, anti-thyroid autoantibody screening, and proper intervention.

## Methods

### Subjects

Women in early pregnancy diagnosed as SH in the outpatient clinic of the endocrinology department of Tianjin Medical University General Hospital from September 2014 to October 2016 were included in the study. The control group comprised those in early pregnancy diagnosed as euthyroidism (EH) in the same hospital. Gestational age was determined from foetal B mode ultrasonography and the time of the last menstrual period. All subjects were local residents and had lived in Tianjin more than 5 years. Women with history of thyroid diseases or other comorbidities and women who took thyroid medications, anti-thyroid drugs, iodine-containing medications or supplements, or iodinated contrast agents (e.g. amiodarone, cydiodine buccal tablets, intravenous contrast agents, and iodine-containing nutrient supplements) within the past 3 months were excluded from the study.

The study was conducted in accordance with the principles of good clinical practice and the Declaration of Helsinki. The study protocol was approved by The Ethics Committee of Tianjin Medical University General Hospital, and written informed consent was obtained from all participants.

### Sample collection

Five millilitres fasting venous blood, without coagulation, was drawn from all participants. Serum was separated within 6 h and stored at −80 °C. Ten millilitres of fasting urine were collected in specialised containers and stored at −80 °C for testing UIC and urinary creatinine (UCr) concentration.

### Testing of thyroid function

Serum thyroid-stimulating hormone (TSH), free thyroxine (FT_4_), free triiodothyronine (FT_3_), thyroid peroxidase antibody (TPOAb), and thyroid globulin antibody (TGAb) levels were all tested using a chemiluminescence immunoassay test kit from Siemens, Germany. Quality control was performed for all test kits. The intra- and inter-assay coefficients of variation (*n* = 20) in our laboratory were, respectively, 2.7 and 4.6% for FT_3_, 3.2 and 4.9% for FT_4_, 2.6 and 4.6% for TSH, 7.2 and 11.2% for TPOAb, and 7.9 and 12.3% for TGAb.

### Testing of UIC and UCr

UIC was estimated using As^3+^-Ce^4+^ catalytic spectrophotometry (WS/T-2006), a national method developed by China’s Ministry of Health. To exclude the impact of the volume and level of urine concentration on UIC, UCr was tested along with urinary iodine, and the UIC/UCr ratio was calculated.

### Diagnostic criteria

All diagnoses were made according to specified reference values for gestational thyroid function as recommended by the guidelines for the diagnosis and treatment of gestational and postpartum thyroid diseases in China proposed in 2012 [13]. Reference values for TSH, FT_4_, TPOAb, and TGAb were 0.13–3.93 mIU/L, 12.00–23.34 pmol/L, 0–35 IU/L, and 0–20 IU/L, respectively. Participants were divided into EH and subclinical hypothyroidism (SH) groups according to TSH and FT_4_ levels. Women with both normal TSH and FT_4_ were included in the former, while those with TSH levels higher than the upper reference limit and normal FT_4_ values were included in the latter group.

### Gestational iodine nutritional standards

Iodine status was classified according to the World Health Organization (WHO) criteria for pregnant women [14]. Median UIC of <20 μg/L, 20–50 μg/L, 51–149 μg/L, 150–249 μg/L, 250–499 μg/L, and ≥500 μg/L corresponded to severe, moderate, and mild iodine deficiency, and adequate, more-than-adequate, and excessive iodine intake, respectively.

### Ultrasonography

Thyroid ultrasonography was performed by the same experienced physician using commercially available high-definition colour-Doppler ultrasound equipment (HD11, Philips Healthcare, Netherlands) equipped with a 7.5–12 MHz high frequency linear transducer. Patients were examined in a supine position with their neck hyperextended in accordance with a standard sonographic protocol. The volume of each lobe was calculated separately, using a formula for elliptical shape volume [(π/6) × length × width × depth]. The thyroid volume was a sum of the volumes of both lobes [15].

### Statistical analysis

Data were analysed using SPSS 19.0 software (IBM, US). Normally distributed data (age and gestational weeks) were reported as mean ± standard deviation, and skewed data (FT_3_, FT_4_, TSH, UIC, and UIC/UCr) were presented as median and interquartile range (25^th–^75^th^ percentiles). Two independent-sample *t*-tests were used to compare age and gestational weeks, and Wilcoxon rank sum tests were used to compare FT_3_, FT_4_, TSH, UIC, and UIC/UCr between the two groups. The chi-squared test or Fisher’s exact test was adopted for frequency comparison. Spearman rank bivariate correlation was adopted for correlation analysis. Two-tailed cut-offs were used, and a *P* value <0.05 was considered statistically significant.

## Results

### Demographic features of patients

A total of 219 women in early pregnancy (average 10.1 ± 2.2 weeks) with an average age of 28.3 ± 3.1 years were enrolled in the study. One hundred four subjects in the EH group were aged 28.0 ± 3.0 years and had gestated for 10.1 ± 2.3 weeks, while 115 subjects in the SH group were aged 28.6 ± 3.2 years and had gestated for 10.2 ± 2.0 weeks. There were no significant differences between age and gestational week between the two groups (Table 1).

**Table 1:** Comparison of baseline characteristics, urinary iodine concentration, and prevalence of anti-thyroid peroxidase antibody and anti-thyroid globulin antibody between the euthyroidism and subclinical hypothyroidism groups

Group	n	Mean age	Mean number of gestational weeks	Median TSH (μIU/mL)	Median FT_4_ (pmol/L)	Median FT_3_ (pmol/L)	Median UIC (μg/L)	Median UIC/UCr (μg/g)	Prevalence of TPOAb (%)	Prevalence of TGAb (%)
Euthyroidism	104	28.0 ± 3.0	10.1 ± 2.3	2.22 (1.44–2.82)	15.03 (13.76–16.67)	4.83 (4.45–5.13)	150.1 (103.5–187.5)	131.7 (82.8–183.9)	56.7%	40.4%
Subclinical hypothyroidism	115	28.6 ± 3.2	10.2 ± 2.0	5.81 (4.70–7.67)	14.30 (12.93–15.36)	4.61 (4.37–5.03)	154.0 (93.1–243.1)	140.7 (98.1–226.4)	79.1%	69.6%
P		0.161	0.160	0.000	0.002	0.074	0.231	0.143	0.000	0.000

*TSH* Thyroid-stimulating hormone, *FT4* Free thyroxine, *FT3* Free triiodothyronine, *UIC* Urinary iodine concentration, *UCr* Urinary creatinine, *TPOAb* Anti-thyroid peroxidase antibody, *TGAb* Anti-thyroid globulin antibody

### Iodine nutritional status in early pregnancy with SH in Tianjin

**Fig. 1:**
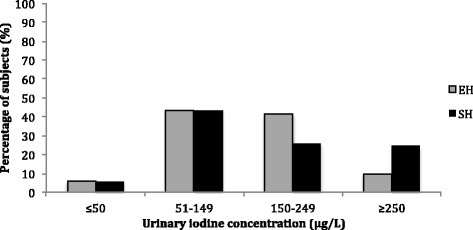
Urinary iodine concentration (UIC) distribution among early pregnant women with euthyroidism and subclinical hypothyroidism in Tianjin. The percentage distribution was significantly different between the euthyroidism (EH) and subclinical hypothyroidism (SH) groups (*χ*2 = 10.656, *p* = 0.014). The percentage of those with UIC ≥ 250 μg/L in the SH group was significantly higher than that in the EH group (*χ*2 = 8.265, *p* = 0.004)

The median (range) UIC in the SH and EH groups was 154.0(93.1–243.1) μg/L and 150.1(103.5–187.5) μg/L, respectively, with no significant difference between the groups (Z = −1.198, *p* = 0.231). The median UIC/UCr ratio in the SH and EH groups was 140.7(98.1–226.4) μg/g and 131.7(82.8–183.9) μg/g, respectively, and was not significantly different between the groups (Z = −1.465, *p* = 0.143) (Table 1). Among those in the SH group, the distribution by UIC ≤ 50 μg/L, 51–149 μg/L, 150–249 μg/L and ≥250 μg/L levels were 6.0, 43.5, 26.2, and 24.3%, respectively, while those in the EH group were 5.8, 43.3, 41.3, and 9.6% respectively. There was a significant difference in the percent distributions between the two groups (*χ* ^2^ = 10.656, *p* = 0.014). The percentage of those with UIC ≥ 250 μg/L in the SH group was significantly higher than that in the EH group (*χ* ^2^ = 8.265, *p* = 0.004) (Table 2, Fig. 1).

**Table 2:** Comparison of urinary iodine concentration (UIC) frequency distribution between the euthyroidism and subclinical hypothyroidism groups

Group	UIC (μg/L) frequency distribution				Total
	≤50	51–149	150–249	≥250	
Euthyroidism	6	45	43	10	104
Subclinical hypothyroidism	7	50	30	28	115
Total	13	95	73	38	219
P	0.921	0.975	0.017	0.004	0.014

### Prevalence of TPOAb and TGAb and its relationship with thyroid function

The prevalence of TPOAb and TGAb in the SH group was significantly higher than that in the EH group (79.1% vs 56.7%, *χ* ^2^ = 12.697, *p* = 0.000 for TPOAb and 69.6% vs 40.4%, *χ* ^2^ = 18.847, *p* = 0.000 for TGAb). FT_4_ level in the SH group was significantly lower than that in the EH group (Z = −3.157, *p* = 0.002), however FT_3_ and thyroid volume did not differ significantly between the two groups. There was no significant relationship between TSH or FT_4_ and TPOAb level in women in early pregnancy (*p* > 0.05).

### UIC and ultrasonographic features of subjects in the SH group with negative TPOAb and TGAb

Among subjects in the SH group, 15.7% were negative for both TPOAb and TGAb. In the SH group, there were no significant differences in TSH and FT_4_ between the autoantibody-negative and -positive subgroups (*p* = 0.706 for TSH and *p* = 0.122 for FT_4_). To explore the possible aetiology of SH in subjects with negative anti-thyroid autoantibodies, we analysed UIC and ultrasonographic features of the subjects. The distribution frequencies in the autoantibody-negative SH group among those with UIC < 150 μg/L, 150–249 μg/L and ≥250 μg/L were 38.9% (7/18), 33.3% (6/18), and 27.8% (5/18), respectively, while those in the autoantibody-negative EH group were 50.0% (20/40), 45.0% (18/40), and 5.0% (2/40), respectively. The distribution frequencies tended to be different between the two groups, but the difference was not statistically significant (*p* = 0.066) (Table 3). The percentage of those with more-than-adequate and excessive iodine in the autoantibody-negative SH group tended to be higher than that in the autoantibody-negative EH group, but the difference was not statistically significant (*p* = 0.025, adjusted test level α = 0.0167) (Table 3). In the autoantibody-negative SH group, eight of 18 presented as diffuse hypoechoic and heterogeneous echo texture by ultrasonography, and were thereby diagnosed as Hashimoto thyroiditis.

**Table 3:** Comparison of urinary iodine concentration (UIC) frequency distribution between the euthyroidism and subclinical hypothyroidism groups with negative anti-thyroid autoantibodies

Group	UIC (μg/L) frequency distribution			Total
	<150	150–249	≥250	
Euthyroidism	20	18	2	40
Subclinical hypothyroidism	7	6	5	18
Total	27	24	7	58
P	0.433	0.404	0.025	0.066

### The relationship between UIC and thyroid function in early pregnancy

UIC was not significantly correlated with FT_3_, FT_4_, and TSH levels in early pregnancy (*p* > 0.05).

## Discussion

Demand for iodine as a nutrient increases in pregnancy to support a series of physiological changes [16, 17], including increased iodine clearance as a consequence of increased renal blood flow and glomerular filtration, transplacental iodine transfer from mother to foetus, increased demand for thyroid hormone due to maternal hormone transplacental transfer to foetus, and increased combined thyroid hormone due to increased thyroid hormone-binding globulin production in response to higher oestrogen levels. Severe iodine deficiency in pregnancy can lead to maternal hypothyroidism, impairing neurological development of the foetus [18]. Moreover, even mild and moderate iodine deficiency in early pregnancy can impair the final intelligence and scholastic ability of progeny [19, 20]. On the other hand, excessive iodine intake may be detrimental to pregnant women and increase their risk for developing hypothyroidism and autoimmune thyroiditis [21]. Investigations such as the current study that investigate iodine nutrition status in women with SH in early pregnancy may help to provide a basis for proper iodine intake guide.

As >90% of ingested iodine is ultimately excreted in the urine, UIC is a good indicator of iodine nutritional status, and median UIC from a large sample in a previous study has been widely used as a biomarker of population iodine intake [22]. WHO recommends an optimum median UIC of 150–249 μg/L for pregnant women [14]. In the current study, median UIC in early pregnant women with subclinical hypothyroidism and euthyroidism was 154.0(93.1–243.1) and 150.1(103.5–187.5) μg/L, respectively, both of which are at the lower limit of the iodine sufficiency range. In contrast, results of iodine nutrition status surveys in different geographical regions of China have produced disparate results. In Henan province, an inland area, median UIC in pregnancy was 198 μg/L, and the iodine nutrition was adequate [23], while in Zhejiang province, a coastal area, median UIC in pregnancy was 130 μg/L, and the iodine nutrition was deficient [11]. In Shijiazhuang province, median UIC declined as gestational age increased, and the iodine nutrition status shifted from iodine sufficiency in the first trimester to deficiency in the second and third trimesters of pregnancy [24]. The present study showed that iodine nutrition status in early pregnancy in Tianjin was adequate, but that median UIC was at the lower limit; in addition, at mid-gestation, the foetal thyroid gland begins to produce thyroid hormone, leading to transplacental iodine transfer, further exacerbating iodine deprivation of the mother; accordingly, pregnant women in Tianjin are likely still at risk of iodine deficiency as the pregnancy progresses. According to WHO, pregnant women should have an iodine intake of at least 250 μg daily [14]. Since the iodine content of edible salt in Tianjin is 30 mg/kg (permitted range ±30%) [25] and the average salt consumption is inadequate at 6 g/d (the average reported consumption), pregnant women in Tianjin require additional iodine intake from other sources.

The current study showed that the percentage of those with more-than-adequate and excessive iodine in the SH group was significantly higher than that in the EH group. These findings indicate that iodine excess, rather than iodine deficiency, is associated with SH in early pregnancy in Tianjin. In most cases, excessive iodine intake may inhibit thyroid hormone synthesis and secretion to protect the body from thyrotoxicosis, the so-called acute Wolff-Chaikoff effect [26], which is typically transient and lasts only 24–48 h. However, some vulnerable individuals with underlying thyroid abnormalities might fail to escape from the Wolff-Chaikoff effect, resulting in iodine-induced hypothyroidism [27]. Secondly, iodine excess itself can trigger autoimmune thyroiditis in some genetically susceptible individuals and lead to hypothyroidism. A study conducted by Sang et al. revealed that excessive iodine intake during late pregnancy might lead to maternal thyroid dysfunction, particularly SH [21]. A cross-sectional study of 7190 women in early pregnancy in China also showed that UIC ≥ 250 μg/L is associated with a significantly high risk of SH and that UIC ≥ 500 μg/L is associated with a high risk of isolated hypothyroxinemia [8]. Therefore, pregnant women with SH should be instructed to consume an appropriate amount of iodine to avoid excessive iodine intake.

The prevalence of TPOAb (79.1%) and TGAb (69.6%) positivity in early pregnancy with SH was significantly higher than that in subjects with euthyroidism, suggesting that autoimmune thyroiditis is the major cause of SH in early pregnancy in Tianjin. Additionally, TPOAb and TGAb positivity is a risk factor for postpartum thyroiditis [28] and thus deserves particular attention in pregnant women.

Although 15.7% of subjects in the SH group were both TPOAb- and TGAb-negative, the serum TSH and FT_4_ level was not significantly different between the autoantibody-negative and -positive subgroups. Eight of 18 subjects in the SH group with negative thyroid antibodies were diagnosed as autoimmune thyroiditis by means of thyroid ultrasonography. Rago has confirmed the value of thyroid ultrasonography in detecting autoimmune thyroiditis in cases with negative thyroid antibodies [29]. Therefore, SH with negative anti-thyroid autoantibodies in early pregnancy in the present study might partly be attributable to serological negative autoimmune thyroiditis. Moreover, there tended to be a greater percentage of those with more-than-adequate and excessive iodine in the autoantibody-negative SH group than that in the autoantibody-negative EH group, therefore more-than-adequate and excessive iodine, rather than iodine deficiency, might be associated with SH in those with negative thyroid autoantibodies. However due to the small sample size, these findings need to be further investigated.

There were remaining ten subjects both negative for thyroid antibodies and ultrasonography, in the literature, such cases are referred to as “idiopathic subclinical hypothyroidism” [30] or “non-autoimmune subclinical hypothyroidism” [31]. Recently, idiopathic SH was reported to be associated with TSH resistance due to TSH receptor mutations [31]. Furthermore, obesity and overweight are related to elevated TSH level [32], but the contribution of elevated TSH level to SH in pregnancy remains unclear and needs to be investigated.

There are some limitations in our study. Firstly, the incidence of SH in pregnancy has been reported to be 2–4% [6], and some pregnant women do not undergo thyroid function screening in early pregnancy. To achieve a sample size of approximately 100, we screened 4600 women in early pregnancy, thus there was a small sample size. Secondly, the spot UIC used to assess iodine nutrition status of the subjects only reflects recent iodine intake and varies according to extent of hydration, thus limiting its usefulness as a screening tool.

## Conclusions

In summary, the current results show that women with SH in early pregnancy in Tianjin were iodine sufficient, but remained at risk of iodine deficiency as pregnancy progressed. Moreover, more-than-adequate and excess iodine (UIC ≥ 250 μg/L) were associated with SH. In addition, serological negative autoimmune thyroiditis and UIC ≥ 250 μg/L may play a role in pathogenesis of SH with negative autoantibodies. However, owing to the small sample size and limited usefulness of spot UIC, a larger sample study is needed to confirm the results.

## Abbreviations

EH: Euthyroidism
FT_3_: Free triiodothyronine
FT_4_: Free thyroxine
SH: Subclinical hypothyroidism
TGAb: Anti-thyroid globulin antibody
TPOAb: Anti-thyroid peroxidase antibody
TSH: Thyroid-stimulating hormone
UCr: Urinary creatinine
UIC: Urinary iodine concentration
WHO: World Health Organization
